# Symptom Burden among Hospitalised Older Patients with Heart Failure in Hanoi, Vietnam

**DOI:** 10.3390/ijerph192013593

**Published:** 2022-10-20

**Authors:** Thanh Thi Nguyen, Thanh Xuan Nguyen, Thu Thi Hoai Nguyen, Tam Ngoc Nguyen, Huong Thi Thu Nguyen, Huong Thi Thanh Nguyen, Anh Trung Nguyen, Thang Pham, Huyen Thi Thanh Vu

**Affiliations:** 1 Department of Geriatrics, Hanoi Medical University, Hanoi 100000, Vietnam; 2 Scientific Research Department, National Geriatric Hospital, Hanoi 100000, Vietnam; 3 Dinh Tien Hoang Institute of Medicine, Hanoi 100000, Vietnam; 4 Physiology Department, Hanoi Medical University, Hanoi 100000, Vietnam

**Keywords:** heart failure, symptom burden, Vietnam, palliative care

## Abstract

This study aimed to assess the symptom burden among older patients hospitalised for heart failure. This hospital-based, cross-sectional study was conducted at the National Geriatric Hospital, Hanoi, Vietnam, from June 2019 to August 2020. Face-to-face interviews were performed to gather the following information: socio-demographic characteristics, heart failure classification, and clinical characteristics (comorbidities, polypharmacy, pro–B-type natriuretic peptide, left ventricular ejection fraction (LVEF), symptom burden, and depression). Symptom burden was assessed using the Edmonton Symptom Assessment Scale (ESAS), and depression was measured using the Patient Health Questionnaire. A total of 314 patients participated in the study. The mean participant age was 72.67 (SD = 9.42) years. The most frequently reported symptoms on the ESAS were shortness of breath (95.5%), fatigue (94.8%), and anxiety (81.2%). In univariate analyses, depression was significantly associated with heart failure class (*p* < 0.05). Multivariate linear regression revealed that major depression was significantly associated with total symptom burden score (Beta: 11.74; 95% CI: 9.24–14.23) and LVEF (Beta: −0.09; 95% CI: −0.17–(−0.007)). Patients hospitalised for heart failure experienced a high burden of symptoms. Further studies addressing adverse outcomes and expanding to community-dwelling older people are essential. Palliative care approaches that target symptom reduction should be considered in patients with heart failure.

## 1. Introduction

Heart failure (HF) is a major and growing public health problem among older people that leads to increased mortality, morbidity, and hospitalisation. An estimated 64.3 million people worldwide live with HF [1]. HF is an age-related syndrome; in men, its prevalence increases from 6.6% in patients aged 66–69 years to 10.6% in those aged 80 years or older [1]. HF is a complex clinical syndrome that is associated with reduced quality of life and functional ability, significant morbidity and mortality, and high costs to patients, health care systems, and society [2,3,4,5].

Patients hospitalised for HF present with a myriad of symptoms. In addition to classic symptoms such as dyspnoea, oedema, and fatigue, previous research has shown that patients often experience fewer specific symptoms, including pain, anorexia, anxiety, and depression [6]. Depressive symptoms may predict higher rates of mortality and have been associated with the frequency and severity of symptoms in patients with HF [7,8]. The primary goals of HF treatment include reducing symptom burden, minimising disease progression, and enhancing health-related quality of life [6]. Appropriate knowledge of symptom burden can inform clinicians in managing symptoms, identifying the long-term effects of therapies, and providing optimal care.

The rate of HF hospitalisation is increasing in Vietnam [7]. However, limited published studies have investigated symptom burden in hospitalised older people with HF. Therefore, this study assessed symptom burden and its related factors among older patients hospitalised for HF at the National Geriatric Hospital in Vietnam.

## 2. Materials and Methods

### 2.1. Study Population

This cross-sectional study was conducted at the National Geriatric Hospital, Hanoi, Vietnam, from June 2019 to August 2020. Patient selection was performed through a review of the medical record. Patients were invited to participate in the study if they met the following criteria: (1) age of 60 years or older, (2) hospitalised for at least 3 days at the time of the interview, and (3) diagnosed with HF based on the European Society of Cardiology 2016 diagnostic criteria [9]. Patients were excluded from the study if they were unable to complete the study’s assessments: patients had severe conditions (defined as dying or receiving intensive care), were blind or deaf, or had dementia or severe cognitive impairment. Convenience sampling was employed, and all patients admitted to the hospital with HF were invited to participate in the study. Of 395 patients with HF admitted to the hospital, 54 (0.13%) refused to participate, 19 (0.04%) were unable to provide informed consent, and 8 (0.02%) did not complete the study assessments. A total of 314 participants were thus enrolled in this study. 

### 2.2. Data Collection

Face-to-face interviews in the Vietnamese language were performed by a physician and five well-trained nurses at the National Geriatric Hospital using a structured questionnaire. Before conducting data collection, the study’s objectives were thoroughly explained to all participants, and the data collectors completed a training program on screening and data collection. The questionnaire gathered the following information.

Socio-demographic information: information regarding age, gender (male/female), education level (high school or less/more than high school/illiterate), and caregiver status (self-care/caregiver) were collected.

#### 2.2.1. Clinical Characteristics

The comorbidities assessed included hypertension, coronary artery diseases, valvular heart disease, atrial fibrillation, diabetes, chronic kidney diseases, chronic obstructive pulmonary disease, and pneumonia. Comorbidities were obtained from the medical records and interviews. Patients’ medications at discharge were collected from the medical records and prescriptions.

HF was classified according to symptom severity using the New York Heart Association (NYHA) functional classification [10,11]. Patients were placed in one of four categories based on their level of physical activity limitation.

Blood samples were taken and centrifuged within 2 h of collection to obtain a pro–B-type natriuretic peptide (Pro-BNP) level. The left ventricular ejection fraction (LVEF) was collected from the prior echocardiogram result with a cut-off of 50% [9]. The equations used to determine left ventricular (LV) and to perform the test were carried out by using a Vivid S70 machine (made in China and manufactured in 2017).

#### 2.2.2. Symptom Burden

Symptom burden was assessed using the Edmonton Symptom Assessment Scale (ESAS), a ten-question symptom survey that has been validated in HF populations [12]. The questionnaire evaluates the presence and severity of ten symptoms, pain, fatigue, drowsiness, nausea, lack of appetite, shortness of breath, depression, anxiety, well-being, and others (we chose oedema in this study), using a numeric rating scale (from 0 to 10 points, with 0 representing the best and 10 representing the worst possible health condition). The ESAS symptoms were categorised into the following four categories of severity: none (0 points), mild (1–3 points), moderate (4–6 points), and severe (7–10 points). The total symptom burden score is the sum of the ten symptom scores and ranges from 0 to 100. High scores indicate greater symptom burden [12]. The ESAS has good reliability and validity, with a Cronbach’s α coefficient of 0.68 and a split-half coefficient of 0.57.

Depression was measured using the Vietnamese version of the Patient Health Questionnaire (PHQ-9) [13]. The PHQ-9 is based on the Diagnostic of Mental Disorders diagnostic criteria for clinical depression and has been validated and demonstrated to have acceptable psychometric properties in patients with HF [14,15]. Patients were asked to rate each of the nine items based on how often they were bothered by each symptom over the previous 2 weeks. The items have a 4-point response scale of 0 (‘not at all’), 1 (‘several days’), 2 (‘more than half the days’), and 3 (‘nearly every day’). Possible scores range from 0 to 27, with higher scores indicating a higher likelihood of depression. A PHQ-9 score of ≥10 was considered as the cut-off point for major depression, which has been shown to have a sensitivity of 70% and specificity of 92%. 

### 2.3. Ethics Approval and Informed Consent 

Ethical approval was obtained from Hanoi Medical University (NCS 14/HMU-IRB). All participants provided informed consent to participate in the study. This study was compiled in accordance with the Declaration of Helsinki.

### 2.4. Statistical Analysis

Data were managed using Redcap and analysed using SPSS 22.0 (Chicago, IL, USA). Continuous data variables (age, Pro-BNP level, and ESAS score) were presented as mean and standard deviation (SD). Categorical variables were presented as frequency and percentage. A chi-squared test was performed to compare categorical variables between groups. Multiple linear regression analysis was performed to determine independent associations between the total symptom burden score (ESAS score) and gender, caregiver status, education level, number of diseases, number of medications, LVEF, and depression score. Stepwise forward selection strategies were used to produce the reduced model. These strategies selected variables for the final multiple linear regression models using the threshold *p*-value of 0.2. Differences were considered statistically significant when *p* < 0.05.

## 3. Results

### General Characteristics

A total of 314 patients participated in the study. The mean participant age was 72.67 (SD = 9.42) years. The ratio of males to females was 1:1. Nearly half (45.2%) of participants had an LVEF < 40%, and 74.2% were classified as NYHA classes III and IV. The most common comorbidities were hypertension (63.7%), diabetes (29.9%), and coronary artery disease (41.4%). The most frequently prescribed medications were diuretics (81.5%) and angiotensin-converting enzyme inhibitors and/or angiotensin receptor blockers (64.3%). Only 1.0% of patients were prescribed antidepressant medications (Table 1).

The most frequently reported symptoms on the ESAS were shortness of breath (95.5%), fatigue (94.8%), anxiety (81.2%), pain (75.1%), decreased well-being (71.9%), lack of appetite (71.7%), oedema (54.1%), nausea (30.3%), and drowsiness (15.3%). The most common severe symptoms were fatigue (61.1%) and shortness of breath (55.4%) (Table 2).

The PHQ-9 score was less than 10 points in 132 (42.1%) participants and equal to or greater than 10 points in 182 (57.9%) participants. In univariate analyses, the results showed that depression was significantly associated with NYHA class (*p* < 0.05). Patients in NYHA classes III and IV had a higher prevalence of depression (63.7% and 64.6%, respectively) than patients in NYHA class II (46.9%) (Table 3).

Multiple linear regression revealed that major depression was significantly associated with total symptom burden score (Beta: 11.74; 95% CI: 9.24–14.23) and LVEF (Beta: −0.09; 95% CI: −0.17–(−0.007)) (Table 4).

## 4. Discussion

A key finding in this study was that patients hospitalised for HF experienced a high burden of symptoms, particularly depression. Additionally, consistent with previous studies carried out among hospitalised patients, breathlessness and fatigue were the symptoms with the highest prevalence and severity [16,17,18]. Mental health problems, pain, and decreased well-being had high percentages and were much more prevalent than symptoms considered to be typical, such as oedema and breathlessness. Anxiety and decreased quality of life are not generally thought to be caused by HF and are not always recognised as important signals; thus, they often overlap and are undertreated. Health care professionals usually do not perform mental health screenings and assessments during hospital admission, despite the findings of a systematic review that showed anxiety was associated with increased hospitalisation and mortality in HF [19]. Therefore, it is necessary to conduct assessments of depressive symptoms and pain in patients with HF during hospitalisation to improve the quality of treatment.

Almost half of the study participants reported symptoms of depression associated with symptom burden. The prevalence of depression was higher than that reported in the study by Haedtke (47%) [20,21], which might be due to the higher mean age of participants and the hospital-based design of this study. Similar to the findings of previous studies, in this study there was a significant correlation between NYHA class and depressive symptoms, and the prevalence of depression increased with higher NYHA class [15,22,23]. Proposed mechanisms of the link between depression and worsening HF include increased sympathetic tone and reduced parasympathetic tone, increased cortisol levels, higher rates of platelet aggregation, and high levels of inflammatory cytokines [24]. Due to overlapping signs and symptoms, such as fatigue and dyspnea, depressive episodes are frequently overlooked in HF [6,25]. However, the higher the number of symptoms associated with HF, the easier it is to have depression [25].

The findings of this study suggest that more effort is needed to increase awareness of symptom assessment and management in patients with HF in Vietnam. Our survey provides an overview of the variety of symptoms experienced by elderly patients with HF in clinical practice, including physical and psychological symptoms as well as symptoms related to medications and comorbidities. Comprehensive symptom assessment via user-friendly tools is a key component of effective symptom management. In our study, the prevalence of depressive and pain symptoms was significant, though these symptoms were under-recognised and undertreated. Only 1%, 8.9%, and 3.1% of patients were prescribed antidepressants, pain relief (paracetamol), and opioid medications, respectively. This study raises the question of whether collaborative palliative care in HR management improves symptoms. Multiple studies have shown that patients receiving integrative care experience a decrease in symptoms such as pain, depression, and fatigue [26,27]. In Vietnam, while patients with cancer have access to palliative care, patients with HF do not. Thus, the incorporation of palliative care with evidence-based management to reduce symptom burden and improve quality of life and the building of capacity and knowledge to develop a multidisciplinary team in palliative care for patients with HF are critical [28].

Our study findings have essential implications for everyday practice regarding the concepts of symptom burden, comprehensive assessment tools, and the role of palliative care coordination in HF. To the best of our knowledge, this is the first study of symptom burden in hospitalised older patients with HF in Vietnam. The findings from this study additionally provide evidence and epidemiological data for scientific and medical data banks. We believe that our results highlight the unique perspective of the elderly population with HF. However, this was a cross-sectional study, and we did not evaluate the association of symptoms with adverse patient outcomes. Thus, in the future, longitudinal studies should be implemented to evaluate causal relationships. This study was also conducted in a single hospital. Multicentre studies should be conducted to provide more representative data on older adults with HF in Vietnam. Finally, although the ESAS is the most frequently used scale to assess symptom burden, validation studies of the Vietnamese version are lacking. However, this scale showed internal consistency in the participant response items in the current investigation, with a Cronbach’s α coefficient of 0.68 and a split-half coefficient of 0.57. This scale may thus be useful for symptom burden assessment in HF populations, though further validation studies in the Vietnamese population are needed.

## 5. Conclusions

Patients hospitalised for HF experienced a high burden of symptoms. Depression was associated with a higher number of symptoms and a higher NYHA class. Further studies addressing adverse outcomes and expanding to community-dwelling older people are essential. Palliative care approaches that target symptom reduction should be considered in patients with HF.

## Figures and Tables

**Table 1:** General characteristics (n = 314).

Characteristics	Mean	SD
Age, mean (SD)	72.67	9.42
	n	%
Gender, n (%)		
Female	149	47.5
Male	165	52.5
Educational level, n (%)		
High school or less	241	76.8
More than high school	38	12.1
Illiterate	35	11.1
Caregivers		
Cared	212	67.5
Self-care	102	32.5
Comorbidities, n (%)		
Hypertension	200	63.7
Coronary artery disease	130	41.4
Diabetes	94	29.9
Chronic kidney disease	84	26.8
COPD	22	7.0
Acute lung disease (pneumonia)	83	26.4
Valvular heart disease	101	32.2
Atrial fibrillation	66	21.0
Left ventricular ejection fraction		
LVEF < 40%	142	45.2
LVEF 40–49%	36	11.5
LVEF ≥ 50%	136	43.3
Pro-BNP level, mean (SD)	2562.86	5398.53
NYHA class, n (%)		
I	0	0
II	81	25.8
III	168	53.5
IV	65	20.7
Discharge medications, n (%)		
Loop diuretic (furosemide, thiazide)	256	81.5
ACE inhibitor, angiotensin II receptor blocker	202	64.3
B-blocker (metoprolol, bisoprolol, nebivolol)	55	17.5
Aldosterone receptor antagonist (Verospiron, Aldactone)	67	21.3
Digoxin	68	21.7
Heparin	57	18.2
Vitamin K antagonists (sintrom)	54	17.2
Ivabradin (procoralan)	69	22.0
Antidepressants	3	1
Paracetamol	28	8.9
Opiods	10	3.1

**Table 2:** Symptom reported using Edmonton Symptom Assessment Scale (n = 314).

Symptom Reported	Mild n (%)	Moderate n (%)	Severe n (%)	Total n (%)
Pain	51 (16.2)	130 (41.4)	55 (17.5)	236 (75.1)
Fatigue	8 (2.5)	98 (31.2)	192 (61.1)	298 (94.8)
Shortness of breath	28 (8.9)	98 (31.2)	174 (55.4)	300 (95.5)
Nausea	11 (3.5)	48 (15.3)	36 (11.5)	95 (30.3)
Lack of appetite	31 (9.9)	111 (35.4)	83 (26.4)	225 (71.7)
Anxiety	62 (19.7)	127 (40.4)	66 (21.0)	225 (81.2)
Decreased well-being	94 (29.9)	81 (25.8)	51 (16.2)	226 (71.9)
Drowsy	33 (10.5)	14 (4.5)	1 (0.3)	48 (15.3)
Depression	97 (39.9)	72 (22.9)	49 (15.6)	218 (69.4)
Edema	38 (12.1)	87 (27.7)	45 (14.3)	170 (54.1)

**Table 3:** Associations of depression with NYHA functional classes.

Variables	Total n = 314	Non-Depression n = 132	Depression n = 182	*p* Value
NYHA Class I-IV	n (%)	n (%)	n (%)	<0.05
I	0(0)			
II	81 (25.8)	43 (53.1)	38 (46.9)	
III	168 (53.5)	66 (39.3)	102 (60.7)	
IV	65 (20.7)	23(35.4)	42 (64.6)	

**Table 4:** Multiple linear regression between total symptom burden score and related factors.

Variables	Multiple Linear Regression		
	B	95% CI	
		Lower Bound	Upper Bound
Gender	1.74	−0.87	4.341
Caregivers (yes)	0.26	−2.36	2.873
Educational level (illiterate)	0.73	−3.17	4.624
Number of diseases	0.07	−0.89	1.037
Number of medications	−0.20	−0.82	0.426
Major depression (>10+)	11.74 *	9.24	14.23
LVEF	−0.09 *	−0.17	−0.007

* *p* < 0.05.

## Data Availability

The datasets of this study are available from the corresponding author on reasonable request.
